# Effects of climate change and crop planting structure on the abundance of cotton bollworm, *Helicoverpa armigera* (Hübner) (Lepidoptera: Noctuidae)

**DOI:** 10.1002/ece3.5986

**Published:** 2020-01-09

**Authors:** Jian Huang, HongFei Hao

**Affiliations:** ^1^ Institute of Desert Meteorology China Meteorological Administration Urumqi China; ^2^ Central Asian Research Center for Atmospheric Sciences Urumqi China; ^3^ Bachu Meteorological Administration Bachu China

**Keywords:** abrupt change, climate change, population dynamics, relative abundance

## Abstract

The interactions between plants and insects play an important role in ecosystems. Climate change and cropping patterns can affect herbivorous pest insect dynamics. Understanding the reasons for population fluctuations can help improve integrated pest management strategies. Here, a 25‐year dataset on climate, cropping planting structure, and the population dynamics of cotton bollworms (*Helicoverpa armigera*) from Bachu County, south Xinjiang, China, was analyzed to assess the effects of changes in climate and crop planting structure on the population dynamics of *H. armigera*. The three generations of *H. armigera* showed increasing trends in population size with climate warming, especially in the third generation. The relative abundances of the first and second generations decreased, but that of the third generation increased. Rising temperature and precipitation produced different impacts on the development of different generations. The population numbers of *H. armigera* increased with the increase in the non‐*Bacillus thuringiensis* (Bt) cotton‐planted area. Asynchrony of abrupt changes existed among climate change, crop flowering dates, and the phenology of *H. armigera* moths. The asynchronous responses in crop flowering dates and phenology of *H. armigera* to climate warming would expand in the future. The primary factors affecting the first, second, and third generations of moths were *T*_mean_ in June, the last appearance date of the second generation of moths, and the duration of the third generation of moths, respectively. To reduce the harm to crops caused by *H. armigera*, Bt cotton should be widely planted.

## Graphical abstract

A 25‐year dataset on climate, cropping planting structure, and the population dynamics of cotton bollworms was analyzed to assess the effects of changes in climate and crop planting structure on the population dynamics of *Helicoverpa armigera*. The three generations of *H. armigera* showed increasing trends in population size with climate warming, and the relative abundances of the first and second generations decreased, but that of the third generation increased. The population numbers of *H. armigera* increased with the increase in the non‐Bacillus thuringiensis (Bt) cotton‐planted area, and to reduce the harm to crops caused by *H. armigera*, Bt cotton should be widely planted.

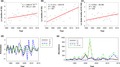

## 1 INTRODUCTION

Biotic and abiotic factors have been shown to affect the population dynamics of birds, mammals, fish, and insects (Sibly, Barker, Denham, Hone, & Pagel, 2005), and understanding density‐dependent and density‐independent processes is central to population fluctuations (Brook & Bradshaw, 2006; Russell et al., 2011; Sibly et al., 2005). Analysis of time series data can provide insights into the relative importance of density‐independent and density‐dependent factors (Berryman & Turchin, 2001). However, the lack of long‐term data on agricultural insect pests limits research aimed at understanding how these factors interact. The cotton bollworm, *Helicoverpa armigera* (Hübner) (Lepidoptera: Noctuidae), exhibits polyphagy, high fecundity, facultative diapause, and high mobility (Downes et al., 2016; Kriticos et al., 2015) and is one of the most serious insect pests in the world (Downes et al., 2016; Fitt, 1989). It usually produces three to seven generations in a year in China, such as three generations in north Xinjiang (Zhang, Ma, Xu, et al., 2013) and Liao River cotton‐planted area (Wu, 1999), four generations in south Xinjiang (Zhang, Ma, Xu, et al., 2013) and north China plain (Zhai, Ding, & Li, 1992), five generations in most area of Yangzi River valley cotton‐planted region and northern area of south China valley cotton‐planted region (Zhang, Li, Ma, & Guo, 2000), and six generations in most area of south China valley cotton‐planted region; in certain years, moths in the regions where it produces six generations can produce seven generations (Meng, Zhang, & Ren, 1962). Wheat serves as the main host of the first generation, while other generations are present on other host crops (Wu & Guo, 2005). China had the highest cotton yield in the world in 2012, with half of this production occurring in the Xinjiang Uygur Autonomous Region (Huang, 2016). Therefore, it is important to understand the drivers of the population dynamics of *H. armigera* in this region.

It has been suggested that elevated CO_2_ under climate change may cause herbivory to increase around two‐ to fourfold and insects to outbreak, while elevated temperature may increase herbivore developmental times, allowing herbivores to partially escape discovery by natural enemies, and drought appears to decimate parasitoid populations (Coley, 1998). Divergences between the thermal preferences of the host and those of the parasitoid lead to a disruption of the temporal or geographical synchronization, increasing the risk of host outbreaks (Hance, Baaren, Vernon, & Boivin, 2007). An organism's environmental tolerance would be better described here as a function of two factors, environmental optimum and genetic contribution to adaptation, and those factors can vary substantially between individuals. Increase of temperature will have a greater negative impact on the distribution of the parasitoid than on its host and that could lead to its exclusion from some agricultural regions where it is currently important (Furlong & Zalucki, 2017). If these changes are beyond their tolerance range, organisms are exposed to stress or lethal conditions (Lynch & Gabriel, 1987). Climate change has been associated with both increased pest outbreaks (Kurz et al., 2008) and decreases in or extinction of insect pest species (Thomas et al., 2004). Climate warming can expand the survival boundary (Franco et al., 2006; Parmesan, 1996; Parmesan et al., 1999; Parmesan & Yohe, 2003), change the phenology (Parmesan, 2007; Satake, Ohgushi, Urano, & Uehimura, 2006; Westgarth‐Smit, Leroy, Collins, & Harrington, 2007), and exacerbate the effect of agricultural intensification on continuously weakening the negative density dependence in regulating the population dynamics of *H. armigera* (Ouyang et al., 2014) and can lessen the negative effects of later spring cold events on these moths and increase their abundance (Gu et al., 2018). Increases in temperature result in higher moth activity and the capture of more moths, and the size of the second generation is significantly related to the size of first generation (Maelzer & Zalucki, 1999; Maelzer, Zalucki, & Laughlin, 1996), although no effects of spring warmth were found (Maelzer & Zalucki, 1999). However, Wardhaugh, Room, and Greenup (1980) indicated that spring warmth could increase the *H. armigera* population in the Narrabri area due to the size of the wheat crop. Furthermore, Maelzer and Zalucki (2000) suggested that the sizes of the first spring generations of both *H. armigera* and *H. punctigera* in Narrabri were significantly correlated with southern oscillation index in certain months, sometimes up to 15 months before the date of trapping.

Exposure to heavy precipitation results in the death of *H. armigera* (Ge, Liu, Ding, Wang, & Zhao, 2003). Precipitation also increases air RH and soil water content. For *H. armigera* pupae, a saturated soil water content results in a lower emergence rate (less than 10%); a soil water content over 60% retards ovary development; a soil water content below 40% promotes the development of the ovary; a soil water content below 20% can improve pupal survival; and a soil water content over 20% decreases egg production in female moths (Chen, Zhai, & Zhang, 2003). Higher RH of 60%–90% is helpful for flight action of adult *H. armigera* (Wu & Guo, 1996). Morton, Tuart, and Wardhaugh (1981) suggested that the flight of *H. armigera* was related to temperature. Precipitation can significantly change the fecundity of *H. armigera* in different generations (Li, Zheng, & Tang, 2016). RH increases with increases in precipitation and leads to abnormal behaviors in insects because the opening and closing of the spiracles of insects result in a discontinuous gas exchange cycle (Chown & Holter, 2000; Hetz & Bradley, 2005). RH mainly affects water within the insect body to produce effects on insects, viz., affecting water balance within the insect body. Water within the insect body is an important induced signal for seasonal action behaviors and can regulate the development and breeding of insects (Tauber, Tauber, Nyrop, & Villani, 1998). Changes in RH can affect water fluctuations in host plants and further impact the feeding of insects, leading to transfer to another region and an increased pest number in that region (Qin, 1964). Precipitation decreases the temperature in summer, which can increase the flight capacity of adult *H. armigera* because the favorable temperatures for its flight have been reported to range from 20°C to 22°C (Gao & Zhai, 2010) or 20°C to 24°C (Wu & Guo, 1996). Suitable temperatures could increase the flight distance of moths and lead to population spreading. Riis and Esbjerg (1998) reported that the flight of the burrowing bug *Cyrtomenus bergi* (Hemiptera: Cydnidae) was also important for population spread. However, Morton et al. (1981) argued that *H. armigera* did not show a significant response to humidity and that the optimum temperature for trapping this species was approximately 27°C. In addition, temperature affects the parasitization rates of *H. armigera*, while RH does not (Kalyebi, Sithanantham, Overholt, Hassan, & Mueke, 2005). However, how the interplay of precipitation and RH impacts the population dynamics of *H. armigera* is unknown.

The climate system is nonlinear and discontinuous. Climate change can be abrupt and can dramatically change from one stable status to another, meaning that climate status varies spatiotemporally from one statistical characteristic to another (Fu & Wang, 1992). Thus, it is necessary to analyze and understand abrupt climate change using nonlinear theories and methods, such as the theory of abrupt changes and corresponding detection methods (Yan, Deng, & Chen, 2003). The Mann—Kendall test presents the merits of a broad detection range, a small artificial impact, and a high degree of quantitativeness (Wei, 1999). Therefore, the Mann—Kendall test was used to detect abrupt changes in climate variables in this study. The detailed theories are provided by Fu and Wang (1992) and Wei (2007). Analysis of the effects of abrupt climate change on crops and insects is helpful for understanding changes in population dynamics. Climate change causes the phenology of crops and *H. armigera* to change with different change rates, which results in asynchrony of abrupt changes between crops and the larvae of *H. armigera* and further affects population numbers due to asynchrony between feeding timing and the food supply (Huang & Hao, 2018; Huang & Li, 2017). However, the effects of abrupt climate change on adult moths of *H. armigera* are unknown.

Crop variety and cropping structure (referring to the crop planting areas and percentages in this study) can both affect *H. armigera*. With the increase in the *Bacillus thuringiensis* (Bt) cotton‐planted area in China, outbreaks of *H. armigera* have been suppressed (Wu, Lu, Feng, Jiang, & Zhao, 2008). Bt cotton significantly suppresses the second‐generation population numbers of *H. armigera* (Gao, Feng, & Wu, 2010). Different crop planting patterns significantly affect the population structure of the arthropod community in Bt cotton fields (Guo, Wan, Hu, & Yan, 2007). Similarly, in Bt cotton field landscapes, the population numbers of *H. armigera* in complex landscapes are lower than those in simple landscapes, and the population numbers of *H. armigera* are negatively correlated with the Bt cotton‐planted area (Lu, Pan, Zhang, Li, & Zhang, 2012; Lu, Zalucki, Perkins, Wang, & Wu, 2013). In non‐Bt cotton fields, many crops, such as wheat, cotton, sorghum, and sunflowers, support successive generations of *H. armigera*, and larval survival fluctuates greatly according to the efficacy of spray application (Wardhaugh et al., 1980). The variability in the proportion of suitable non‐Bt breeding habitat or the total area of Bt and suitable non‐Bt habitat over time can increase the overall rate of resistance evolution by causing short‐term surges of intense selection. These surges can be exacerbated when temporal variation causes high larval densities in refuges, and when Bt crops are rare rather than common in the landscape, rapid resistance evolution can occur (Ives et al., 2017). The numbers of larvae killed by Bt depend not on their total numbers but on larvae proportions of the overall population (Ives et al., 2017).

Thus, the objects of this study were to (a) analyze the long‐term population dynamics of *H. armigera*; (b) identify the change trends of the relative abundance of different generations of moths; (c) determine the effects of climate factors and crop structure on population change; (d) ascertain the impacts of abrupt climate change on abrupt crop changes and abrupt moth changes; and (e) quantify the relative contributions of affecting factors to population dynamics.

## 2 MATERIALS AND METHODS

### 2.1 Study sites

Bachu County (38°47′–40°17′ N, 77°22′–79°56′ E, and 1100–1180 m above sea level), with a total area of 21,741.3 km^2^, lies in the western Tarim Basin, Xinjiang Uygur Autonomous Region, China (Zhang & Zhang, 2006), and comprises a town and several villages. The area has a temperate continental arid climate, and the annual temperature, including mean temperature (*T*_mean_), minimum temperature (*T*_min_), maximum temperature (*T*_max_), precipitation, sunshine hours, and mean frost‐free period are 11.9°C, 5.4°C, 19.5°C, 58.9 mm, 2,994 hr, and 255 days, respectively (Zhang & Zhang, 2006). Cotton, corn, and winter wheat are the primary crops. The planting dates of winter wheat, cotton, and corn were generally around 15th October, 6th April, and 21st June, respectively, during the period of 1991–2015. The fields were irrigated. When *H. armigera* occurred, certain fields were investigated. If 100 ha of crops were investigated at these times, only 5 ha were damaged by *H. armigera*; thus, the occurrence rate was 5%. The total crop area was then multiplied by the occurrence rate, and the damaged crop area at the time could be calculated. The sum of the total damaged area was the accumulated damaged area at every time. The method followed the national standard (Standardization Administration of the People's Republic of China, 2009). The areas of cotton, corn, and wheat fields varied from 14,667 to 83,440, 7,658 to 18,000, and 9,142 to 19,081 ha, respectively, during the period of 1991–2015. Additionally, the area damaged by *H. armigera* varied from 133 to 5,553 ha according to the statistical data from the plant protection station in Bachu County. The study area varied from 31,467 to 120,521 ha during 1991–2015. In this study, only trapped adult moths were employed to analyze changes in abundance. The area in which *H. armigera* adult moths were collected depended on the flight distance of moths. Their short‐range movement distance is approximately 100–1000 m (Fitt, 1989), while their long‐range movement distance is reported to be approximately 10–30 km (Xu, Guo, Wu, & Jiang, 2000) or over 300 km (Wu & Guo, 1996). Migrant moths have been shown to employ sophisticated orientation and height‐selection strategies that maximize displacements in seasonally appropriate directions; they appear to have an internal compass and to respond to turbulence features in the airflow (Chapman, Drake, & Reynolds, 2011). A cloud of moths originating from a cropping region remained extant for over 400 km as it drifted downwind (Wolf, Westbrook, Raulston, Pair, & Hobbs, 1990).The area around the center of a lamp with a radius of 20 km is 125,600 ha, and the lamp could theoretically be used to collect the moths within this study area. Therefore, we regarded this study area as the area for collecting moths.

The life history of *H. armigera* in the locality is as follows. Eggs from the third‐generation (G3) moths become larvae and enter the soil in the state of diapausing pupae in autumn to overwinter, and then become an overwintering generation (G0) moth to produce the eggs of the first generation (G1) in the next spring, and G1 moths emerge from G1 pupae (Wu & Guo, 2000). The G1 larvae mainly use wheat as a host in spring, and the second‐generation (G2) and G3 larvae mainly use cotton and corn as hosts (Li, Yao, Zhou, & Wang, 2005).

### 2.2 Weather and *H. armigera* moth survey data

Weather parameters were recorded at the Bachu weather administration, which lies on the edge of the town. Jun‐Sep meant from June to September; thus, the temperature from June to September was expressed as *T*_max_, *T*_mean_, and *T*_min_ in Jun‐Sep. The adult moths were captured by using a black light lamp with a wattage of 20 (made by Jiaduo Technology, Industry and Trade Company Limited, China), which was placed in an open field at 1.5 m above the ground, with no trees or higher buildings surrounding the lamp. The distance between weather station and the lamp was about 300 m. The lamp was turned off after dawn and turned on after dusk from early April to late September during 1991–2015. Every year the lamp was replaced with a new strip lamp. All the set criteria followed the national standards (the State Administration of Quality Supervision Inspection and Quarantine of the People's Republic of China and the Standardization Administration of the People's Republic of China, 2009). Cotton fields, winter wheat fields, corn fields, other crop fields, and vegetable fields surrounded the lamps. The adult moths captured by using the lamp were counted daily, and every generation of *H. armigera* moths was distinguished using standard methods (Lu et al., 2013; Zhang, Ma, Xu, et al., 2013).

### 2.3 Crop flowering phenology

The flowering dates of wheat, cotton, and corn were the dates on which approximately 50% of the crops flowered, and the calendar dates on which the crops reached each new developmental stage were also recorded. Every growth stage was confirmed according to the national standard (Xu & She, 1980). The growth stages of wheat, cotton, and corn were also recorded in a similar fashion. The crop percentages of crop planting area fluctuated with a range of approximately 10% (Figure 6a).

### 2.4 Statistical analysis

Here, a generation meant adults trapped during the period from the first appearance date of adult moths to their last appearance date. Different generations were distinguished by analyzing the patterns of population fluctuation, that is, the number of adults trapped by the light trap during the season (Lu & Baker, 2013; Miao, Guo, Lu, Yu, & Wang, 2006). According to the life history of *H. armigera* in the studied locality, the periods of G0, G1, G2, and G3 varied from 25th April to 27th May, 3rd June to 11th July, 15th July to 7th August, and 7th August to 20th September, respectively. The moth peak referred to the number of moths trapped reaching 50% of total number of moths trapped during every generation. The moth trough referred to the minimum number of moths trapped, which was zero in this study. To compare the changes in moth numbers in every generation of *H. armigera*, a Gaussian function was used to estimate the population density:

(1) $$f\left(t;A,T,\delta \right)=\frac{A}{\delta \sqrt{2\pi }}e^{-{\left(t-T\right)}^{2}/\left(2\delta^{2}\right)},$$

where *t* refers to the days, *A* is the area under the curve estimating the abundance of individuals, *T* is the mean of the distribution representing to the data on maximum abundance, and *δ* is the standard deviation in estimating the duration of a generation (Gao et al., 2010).

The number of adult moths of *H. armgiera* captured in every generation fluctuated dramatically, so the Ln(moth number) value was used to conduct statistical analysis in this study. The trends of the air temperature and Ln(moth number) over time during the study period were analyzed using linear regression. The relationships between Ln(moth number) and climate parameters were determined using Pearson correlation analyses and regression functions. Statistical significance was declared at *p* < .05. These analyses were conducted by the SPSS 17.0 for Windows statistical package (SPSS Inc.).

The population change rate of *H. armigera* (Turchin, 1999) was calculated using the *R*‐function (Berryman & Turchin, 2001), *R*_t_ = Ln(*N*_t_/*N*_t − 1_), where *N*_t_ and *N*_t − 1_ were the total abundance of captured adult moths in years t and t − 1, respectively. *N* referred to G1, G2, G3, or G1–G3. Thus, the *R* value of every generation could be calculated.

Multicollinearity is a common problem in multivariate analysis. Ordinary least squares (OLS) regression increases the risk of rejecting a theoretically sound predictor (Naes & Martens, 1985) and yields unstable results (Field, 2000). Therefore, partial least squares (PLS) regression was employed because PLS allows comparisons between multiple response variables and multiple explanatory variables (Höskuldsson, 1988), resists overfitting, and is better than principal component analysis (PCA) (Land et al., 2011). The PLS approach was used to determine the relative influence of factors such as climate and crops factors. The SPSS 17.0 for Windows statistical package (SPSS Inc.) was used for these analyses.

The nonparametric Mann—Kendall test (MK) was first developed by Mann (1945) and further developed by Kendall (1948) and Gerstengarbe and Werner (1999). The Mann—Kendall test presents the merits of a broad detection range, small artificial impact, and high degree of quantitativeness (Wei, 2007). In this study, the Mann—Kendall test procedure with a 5% significance level was applied to analyze abrupt changes according to Gerstengarbe and Werner (1999).

### 2.5 Cropping structure

The term “cropping structure” referred to the planted area of crops and percentages of the crop planting areas of species such as winter wheat, cotton, and corn. The planted area percentages of winter wheat, cotton, and corn ranged from 14% to 33%, 39% to 62%, and 11% to 24%, respectively, during the period of 1991–2015.

## 3 RESULTS

### 3.1 Climate change rates

**Figure 1:**
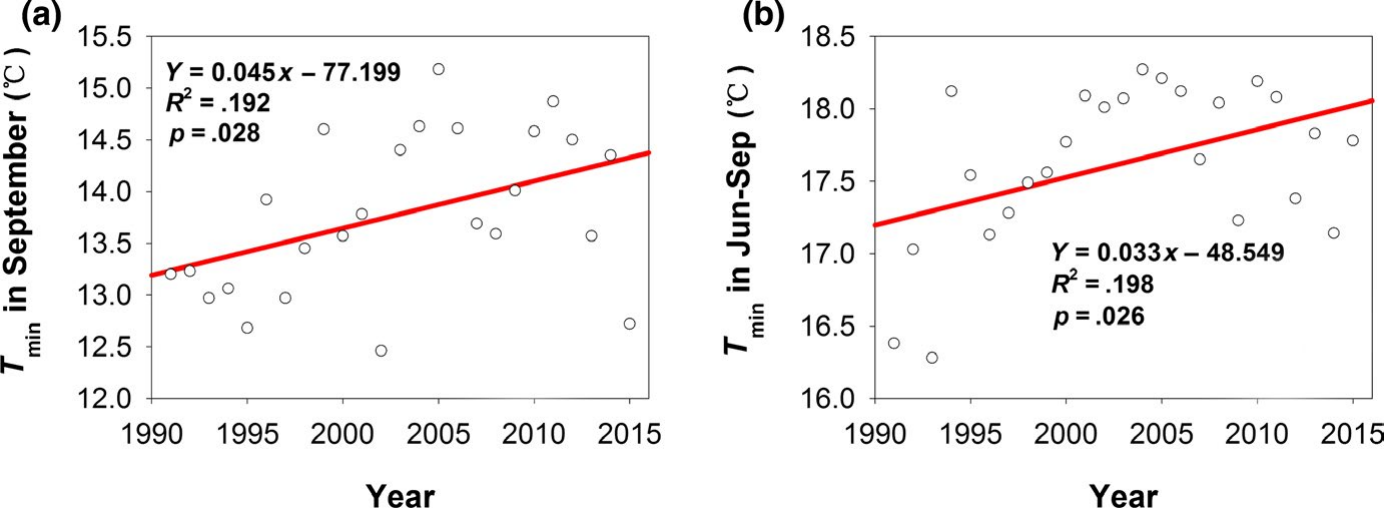
Trends of *T*_min_ in September (a) and Jun‐Sep (b) over time

*T*_min_ in September and Jun‐Sep increased by 0.045°C and 0.033°C per year, respectively, and these changes were significant (Figure 1; Table 1). The increasing and decreasing trends for precipitation, *T*_max_, *T*_min,_ and *T*_mean_ in other months were not significant. Essentially the climate became warmer because the annual *T*_mean_ increased by 0.021°C/year during 1981–2015 (*R* ^2^ = .192, *p* = .008).

**Table 1:** Temperature and precipitation change rates of months

	*T*_min_ (°C/y)	*T*_mean_ (°C/y)	*T*_max_ (°C/y)	Precipitation (mm/y)
May	0.054	0.046	0.063	0.20
June	0.018	−0.001	0.003	−0.23
July	0.012	0.001	0.013	0.28
August	0.056	0.039	0.040	−0.29
September	0.045*	0.007	−0.022	0.33
Summer	0.028	0.013	0.019	−0.79
Jun‐Sep	0.033*	0.012	0.009	0.08

Note

The time series were from 1991 to 2015. * and ** represent significance at *p* < .05 and *p* < .01, respectively. Summer means from June to August. Jun–Sep means from June to September.

### 3.2 Long‐term population dynamics of *H. armigera*

**Figure 2:**
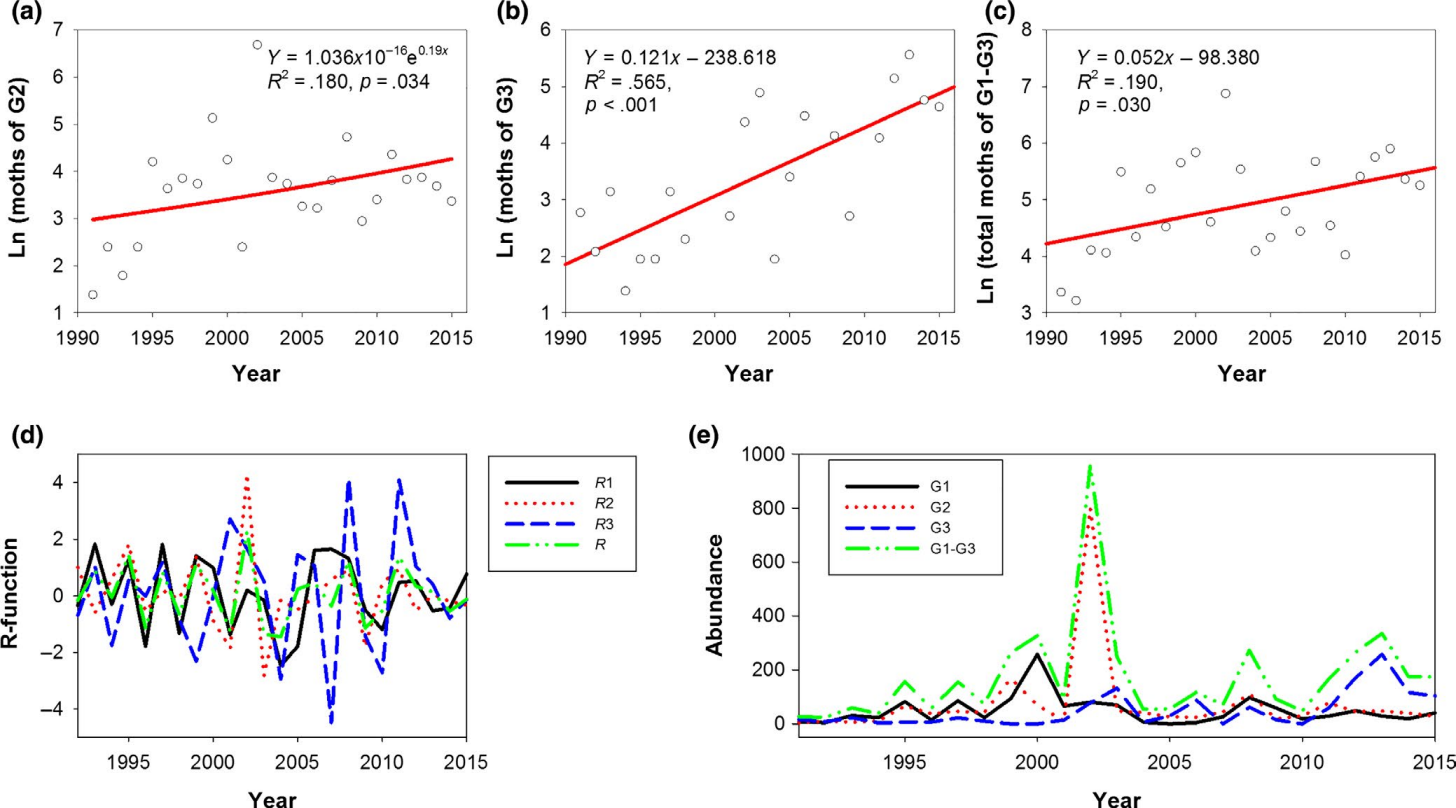
Dynamics of population increase (a, b, c), population change rate (R‐function) (d), and population abundance (e) for *Helicoverpa armigera*

**Figure 3:**
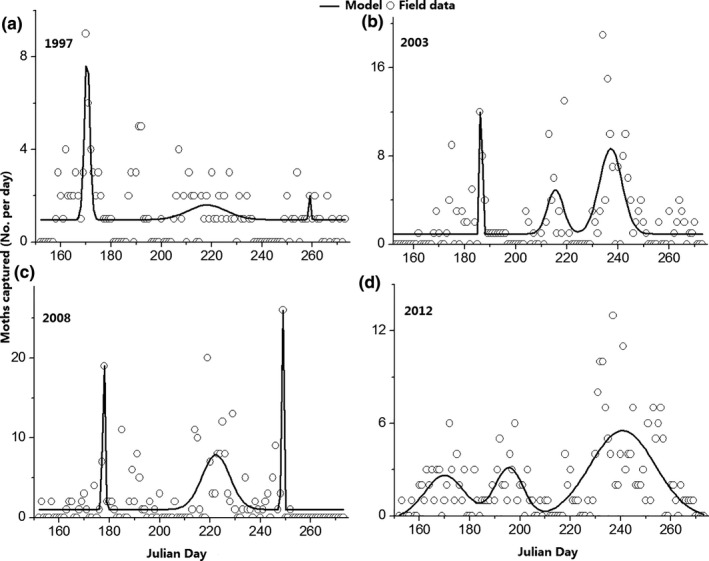
Examples of generation development of *Helicoverpa armigera*. Y‐axis indicated daily light trap catches from 1st June to 30th September in 1997 (a), 2003 (b), 2008 (c), and 2012 (d). The solid black line indicated the fitted population dynamics

Moths of G1 showed an increasing trend in population size over time; however, this was not significant (Y = 0.010*x* − 15.738, *R* ^2^ = .003, *p* = .787). However, moths of G2, G3, and the total numbers of moths of all 3 generations (G1–G3) showed significantly increasing trends in population size over time (Figure 2a‐c). In addition, the results of Gaussian‐fitted curves (Figure 3) showed that the moth peak values of G1, G2, and G3 all showed increased over time; however, only G3 showed a significant increase (Figure 5b). The integral areas under the Gaussian‐fitted curves represented the population numbers. With an increase in the integral area, the population numbers increased (Figure 3).Thus, these results illustrated that both the population numbers and moth peak values of each generation of *H. armigera* at the study site increased from 1991 to 2015.

The population change rates over time of G1, G2, G3, and G1–G3 of *H. armigera* were −0.011, −0.027, 0.018, and −0.013, respectively. However, none of these rates were significant. They all fluctuated near zero, and *R*_3_ exhibited a positive change rate and the greatest fluctuation range (Figure 2d). The abundance of G2 and G1–G3 reached a peak in 2002 (Figure 2e). This finding suggested that the moth numbers fluctuated sharply between years.

### 3.3 Changes in the relative abundance of different generations of *H. armigera* over time

**Figure 4:**
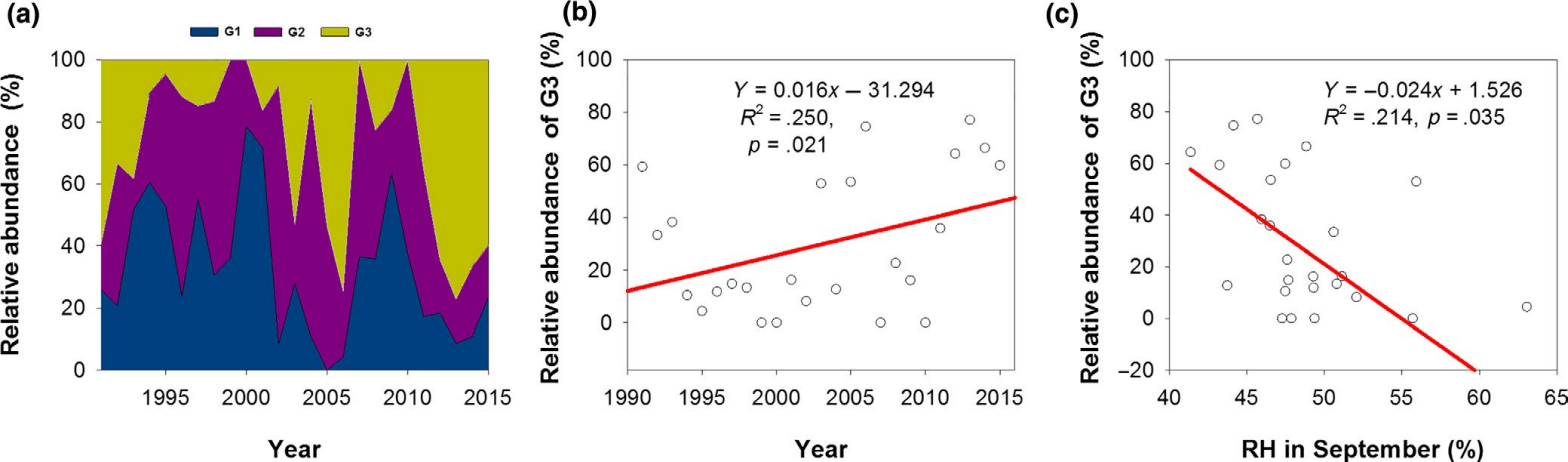
Relative abundance of *Helicoverpa armigera*. Relative abundance of each generation in all three generations (a), increasing trend of the relative abundance of G3 in all three generations (b), and the relationship between the relative abundance of G3 in all three generations and RH in September from 1991 to 2015 (c)

The changes in the relative abundance of different generations of *H. armigera* reflected the changes in the percentages of moth numbers in each generation (Figure 4a). The relative abundance of G1 and G2 showed decreasing trends without significance (data not shown), but the relative abundance of G3 showed a significant increasing trend and increased by 1.6% per year (Figure 4b). Not every generation occurred in each year; for example, G1 was missing in 2005, and a part of G3 was missing in 1999, 2000, 2007, and 2010. The black light lamp did not work for the collection of G3 in 2000 and 2010, and other missing data came from points at which no moths were captured due to mighty wind. The relative abundances of G1and G2 increased from 1991 to 2000, and they decreased from 2000 to 2015. However, the relative abundance of G3 showed an opposite change trend; it decreased before 2000 and increased after 2000 (Figure 4a). The relative abundance of G3 decreased by 2.4% when RH in September increased by 1% (Figure 4c).

### 3.4 Relationships between moth numbers in each generation and climatic factors

**Figure 5:**
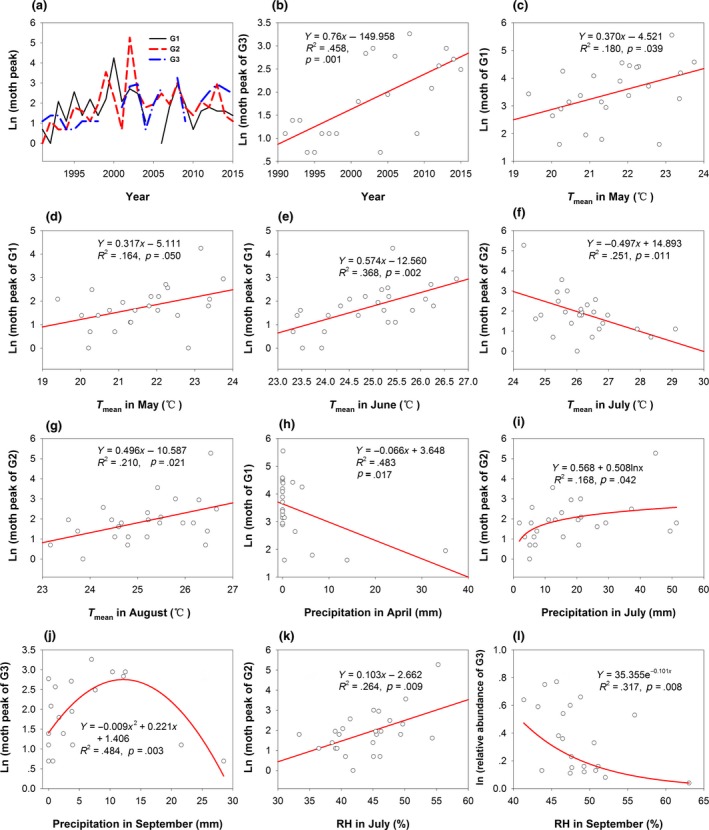
Relationships between climate factors and populations of *Helicoverpa armigera*. Change trends of moth number (a and b), relationships between temperature and moth of G1 (c–e) and G2 (f and g), relationships between moth number and precipitation (h–j), and relationships between moth number and RH (k and l)

The moth peaks of G1, G2, and G3 of 70, 194, and 26 were observed in 2000, 2002, and 2008, respectively (Figure 5a). Ln(moth of G1) significantly increased with *T*_mean_ in May (Figure 5c) and decreased with precipitation in April (Figure 5h). Ln(moth peak of G1) significantly increased with *T*_mean_ in May and June (Figure 5d,e). Ln(moth peak of G2) significantly increased with *T*_mean_ in August and with precipitation and RH in July (Figure 5g,i,k). However, Ln(moth peak of G2) significantly decreased with *T*_mean_ in July (Figure 5f). Ln(moth peak of G3) first increased before precipitation reached 13 mm and then decreased with precipitation in September (Figure 5j). The increase in RH decreased Ln(relative abundance of G3) (Figure 5l).

### 3.5 Effects of cropping structure on *H. armigera* populations

**Figure 6:**
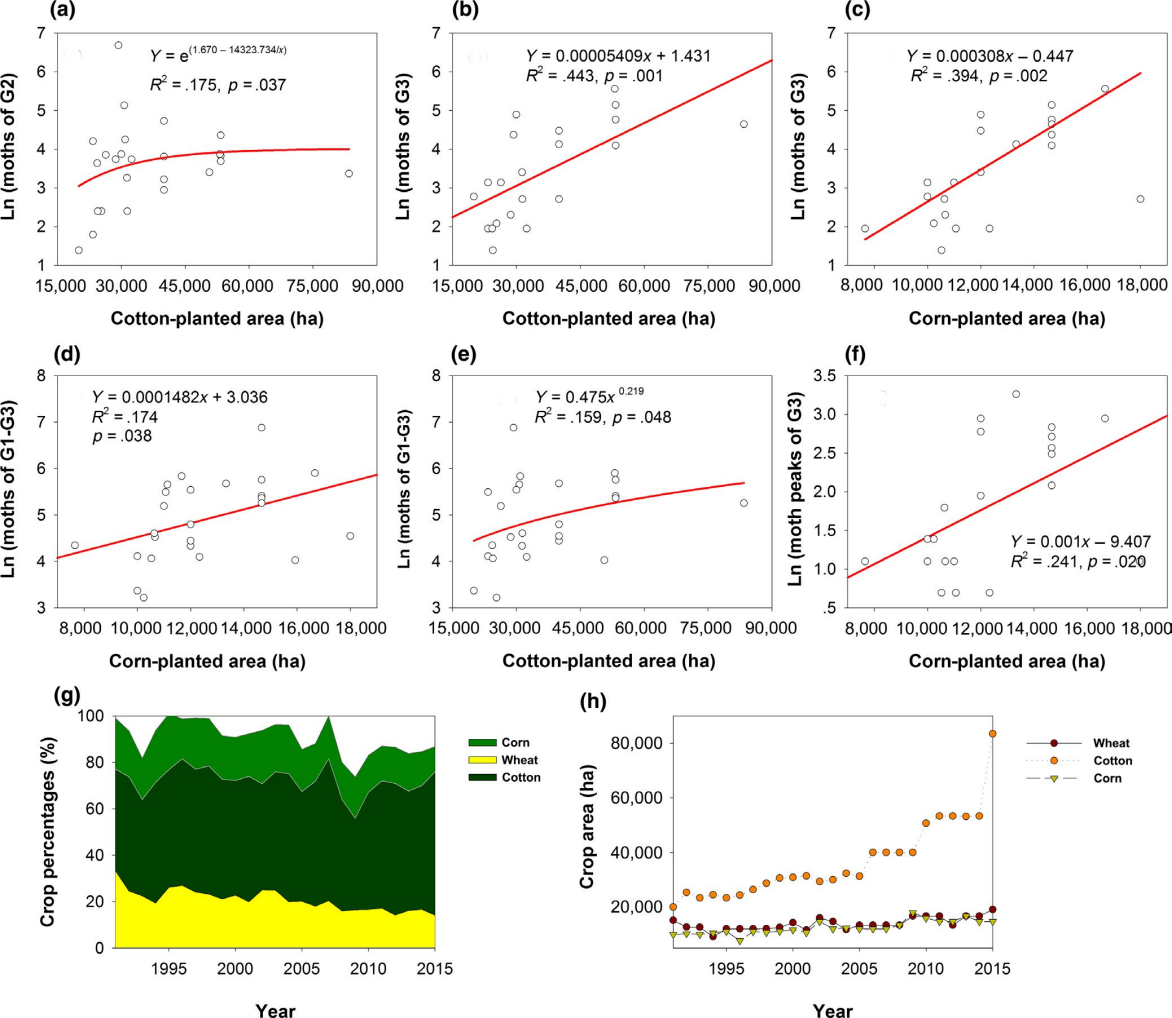
Relationships between crop structure and populations of *Helicoverpa armigera*. Relationships between cotton‐planted area and moths of G2 (a) and G3 (b), relationships between corn‐planted area and moths of G3 (c) and G1–G3 (d), relationship between cotton‐planted area and moths of G1–G3 (e), relationship between moth peaks of G3 and corn‐planted area (f), and change trends of crop percentages (g) and crop area (h)

The Ln(moth numbers) of G2, G3, and G1–G3 significantly increased with the (non‐Bt) cotton‐ and corn‐planted areas (Figure 6a‐e). The cotton‐ and corn‐planted areas increased by 1736.718 and 273.490 ha/year (*p* < .001), respectively. The increase in Ln(G3 moth numbers) associated with the corn‐planted area (Figure 6c) was faster than that with cotton‐planted area (Figure 6b); viz., the moth numbers of G3 increased by 1,000.054 and 1,000.308 per 1,000 ha in cotton‐ and corn‐planted area, respectively. Ln(moth peak of G3) also significantly increased with the corn‐planted area (Figure 6f).

### 3.6 Abrupt changes in climate, moths, and crops

**Figure 7:**
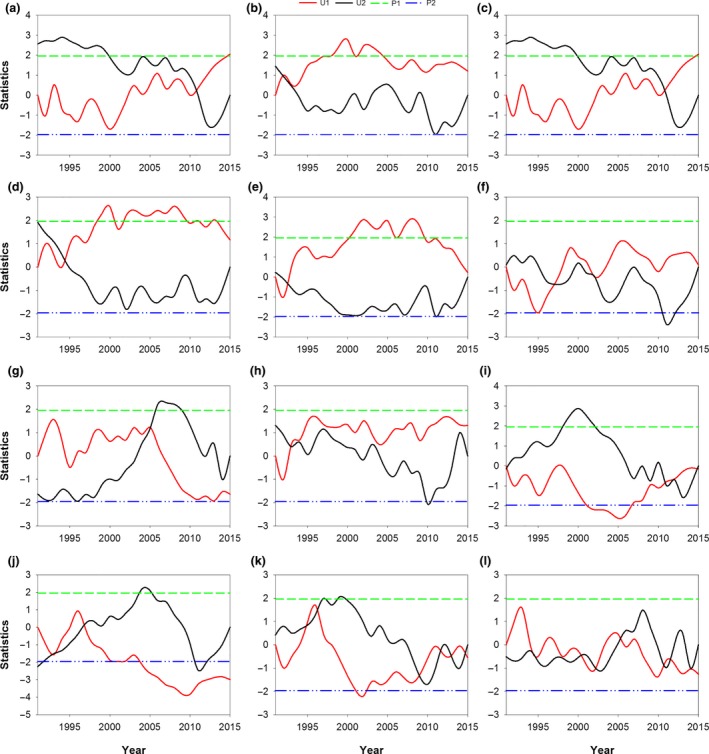
Abrupt changes of moths of G1–G3 (a), moths of G2 (b), moths of G3 (c), peak of G2 moths (d), *T*_mean_ in June (e), *T*_mean_ in September (f), RH in August (g), precipitation in July (h), daily range of temperature in July (i), wheat flowering date (j), cotton flowering date (k), and corn flowering date (l). The four kinds of lines in the figure: a solid red line (*U*_1_), a solid black line (*U*_2_), a dashed green line (*P*_1_), and a dashed blue line (*P*_2_). The *U*_1_ means the forward direction calculated result of the series, and the *U*_2_ means the opposite direction calculated result of the series. The *P*_1_ means the significance level at 5% (*P* = 1.96), and *P*_2_ means the significance level at 5% (*P* = −1.96), viz., the absolute value of *P* (|*P*|) should be |*P*|> 1.96

The results of the Mann—Kendall tests showed significant abrupt changes in the moths of G1–G3 (Figure 7a), moths of G2 (Figure 7b), moths of G3 (Figure 7c), peak of G2 moths (Figure 7d), *T*_mean_ in June (Figure 7e), RH in August (Figure 7g), precipitation in July (Figure 7h), daily range of temperature in July (Figure 7i), winter wheat flowering date (Figure 7j), and cotton flowering date (Figure 7k). The years exhibiting abrupt changes of the ten events mentioned above were 2011, 1993, 2011, 1994, 1992, 2005, 1993, 2012, 1997, and 1996, respectively. An insignificant abrupt change in G1 moths was observed in 1992 (data not shown), and abrupt changes in G2, G3, and G1–G3 moths were observed in 1993, 2011, and 2011, respectively (Figure 7a‐c). Abrupt changes in G1 and G2 moths occurred at the same year, as did those in G3 and G1–G3 moths. In addition, the abrupt changes in G3 and G1–G3 moths exhibited the same change trends (Figure 7a, c). This finding suggested that the abrupt change in G3 moths had a predominant effect on the abrupt change in G1–G3 moths. The abrupt change in *T*_mean_ in June was earlier than those in the moths of G2, G3, and G1–G3, the peak of G2 moths, the winter wheat flowering date, and the cotton flowering date (Figure 7a‐e,j,k). Abrupt changes in G1 and G2 moths were observed in 1992 and 1993, respectively, and abrupt changes in flowering dates of wheat and cotton were observed in 1997 and 1996, respectively. Thus, the abrupt changes in G1 and G2 moths were earlier than those in flowering dates of wheat and cotton. Therefore, the abrupt changes in adult moths, climatic factors, and flowering dates of winter wheat and cotton exhibited asynchrony.

### 3.7 Relative influence ratios of climatic and crop factors on *H. armigera* populations

The moth numbers of G1, G2, G3, and G1–G3 were affected by different factors (Table 2) according to PLS analysis, and the primary affecting factors of G1, G2, G3, and G1–G3 were *T*_mean_ in June, the last appearance date of G2 moths, the duration of G3 moths, and Ln(G3 moth number), respectively. The proportions of variance explained of the four factors ranged from 66.6% to 88.1% (Table 2). The reasons of the four primary affecting factors to different generations were different. G1 moths usually appeared from 3rd June to 11th July, so the increase in *T*_mean_ in June could contribute to producing more moths (Figure 5e). The earlier the first appearance date of G1 moths was, the longer the duration of G1 moths was. In turn, the longer the duration of G1 moths was, the more moths were produced (Y = 0.061*x* + 1.763, *R* ^2^ = .254, *p* = .012). The sum of proportions of variance explained by the first appearance date and duration of G1 moths was 25.9% (Table 2), which could support this observation. G2 moths usually appeared from 15th July to 7th August; *T*_mean_ in July was the highest in a year at this study site, and the higher temperature in July could affect the flight of moths (Figure 5f). Additionally, the increases in precipitation and RH in July contributed to decreasing *T*_mean_ in July and increasing the numbers of G2 moths (Figure 5i,k). Therefore, the later the last appearance date of G2 moths was, the more moths appeared. The sum of proportions of variance explained by the last appearance date and duration of G2 moths was 69.9% (Table 2), which could support this observation. G3 moths usually appeared from 7th August to 20th September, and then, the number of moths significantly increased with prolongation of the duration of G3 moths (Y = 0.067*x* + 1.463, *R* ^2^ = .625, *p* < .001). An earlier first appearance date meant a longer duration of G3 moths. Thus, more moths could develop, which increased the number of G3 moths. The sum of proportions of variance explained by the duration and last appearance date of G3 moths was 79.8% (Table 2), which supports this observation. Thus, the duration of the moths was very important in increasing the numbers of moths, especially for G2 and G3. However, for the total numbers of moths of G1–G3, the number of G3 moths was the primary affecting factor because Ln(G3 moth number) showed a significantly increasing trend (Figure 2b) and the relative abundance of G3 moths increased (Figure 4a), while Ln(G3 moth number) significantly increased with the cotton‐planted area (Figure 6b). In fact, the cotton‐planted area expanded very quickly (Figure 6h). Thus, we could speculate that G3 moth numbers might dramatically increase with expansion of the cotton area, and the total numbers of moths would therefore dramatically increase. Thus, Ln(G3 moth number) had the greatest effect on the total numbers of moths.

**Table 2:** Factors affecting over moths of different generations

Moths number	Factor 1 (%)	Factor 2 (%)	Factor 3 (%)	Factor 4 (%)	Factor 5 (%)	Total (%)
Ln(G1)	A (66.6)	B (23.2)	C (2.7)	D (2.1)	E (1.7)	96.3
Ln(G2)	F (66.8)	G (7.6)	A (4.2)	H (3.1)	I (4.4)	86.1
Ln(G3)	J (77.4)	K (12.9)	L (5.0)	M (2.4)	N (0.6)	98.3
Ln(G1–G3)	O (88.1)	P (6.7)	G (2.2)	N (1.5)	Q (0.6)	99.1

Note

A: *T*_mean_ in June; B: first appearance date of G1 moth; C: duration of G1 moth; D: precipitation in June; E: RH in June; F: last appearance date of G2 moth; G: Ln(G1 moth); H: duration of G2 moth; I: daily range between *T*_max_ and *T*_min_ in July; J: duration of G3 moth; K: wheat planting area; L: cotton planting area; M: first appearance date of G3 moth; N: corn planting area; O: Ln(G3 moth); P: Ln(G2 moth); and Q: precipitation in July.

The analysis of affecting factors for Ln(G1), Ln(G2), Ln(G3), and Ln(G1–G3) was based on the dataset of 1991–2015.

## 4 DISCUSSION

The abundance recorded in this study reached its peak in 2002 (Figure 2e), especially for G2, which caused severe harm to cotton. The population change rates fluctuated near zero both before and after 2002, implying that the population varied from a lower equilibrium to a higher one, though the change rate fluctuated dramatically between years (Figure 2d,e). The abundance of G1–G3 increased from 1991 to 2002 and decreased after 2002, and then increased beginning in 2005 (Figure 2e), which suggested that the crops in this region faced more risks due to the increased moth numbers.

Climate warming has affected the survival, dispersal, development, and abundance of herbivorous insects (Bale et al., 2002; Cornelissen, 2011; Kreyling, 2010), and has shortened the duration of insect development and produces more insects (Liu & Gu, 1997). In this study, *T*_mean_ in Jun‐Sep during the period of 1954–2015 increased by 0.09°C per decade (Y = 0.009*x* + 6.837, *R* ^2^ = .076, *p* = .030). Our results showed that the moth numbers of G1, G2, and G3 all increased over time (Figure 2), and that the durations of G1, G2, and G3 were all prolonged, though this change was significant only for the duration of G3 (Y = 1.073*x* − 2,124.933, *R* ^2^ = .207, *p* = .022) and that a higher in *T*_mean_ in July resulted in lower moth numbers of G2 (Figure 5f). The most suitable temperature for *H. armigera* is 25°C–28°C (Gao, Zhang, Liu, & Wang, 2007), and Noor‐ul‐Ane, Kim, and Zalucki (2018) suggested that the maximum fecundity of *H.armigera* was at 25°C; however, some *T*_mean_ values in July surpassed 28°C, which could cause breeding ability to decrease. Most of the moth peaks were distributed within temperatures of 25°C–28°C (Figure 5f). Liu, Gong, Wu, and Li (2004) indicated that a temperature of over 27°C resulted in summer diapause of *H. armigera* and that summer diapause ratios significantly increased when the temperature reached 33°C.On the other hand, rising RH in July produced more moths (Figure 5k) because the RH in July ranged from 33% to 56%, whereas the most suitable RH for *H. armigera* breeding is 70%–85% (Gao et al., 2007). The temperature in July was the highest within a year, with a mean value of 26.2°C, while the mean RH of 44% was lower because precipitation in July ranged from 5.1 to 51.4 mm, with a mean value of 18.23 mm. Therefore, an increase in RH in July in this arid region could increase the number of moths. Thus, our results suggested that climate warming prolonged the durations of *H. armigera* moths and increased the moth numbers of *H. armigera*.

Precipitation has a great effect on the occurrence of *H. armigera* (Li et al., 2016). In this study, the moth peak of G3 first increased and then decreased (Figure 5j). During the period 1991–2015, a 1 mm increase in precipitation decreased *T*_mean_ in September by 0.042°C (Y = −0.042*x* + 20.701, *R* ^2^ = .288, *p* = .006), which caused *T*_mean_ in September to range from 18.83°C to 21.44°C. Additionally, during the period 1991–2015, the increase in precipitation significantly increased RH in September (Y = 0.336*x* + 46.465, *R* ^2^ = .475, *p* < .001), and the RH decreased *T*_mean_ in September (Y = −0.102*x* + 25.378, *R* ^2^ = .393, *p* = .001). Such temperatures do not suit the development of *H. armigera* because they slow down development, and many pupae will be in diapause when and the photoperiod decreases (Chen, Chao, & Liu, 2016; Hackett & Gatehouse, 1982) and the air temperature is below 20°C (Wu & Guo, 2005) However, the precipitation in July increased the moth peak (Figure 5i). The reason for this increase was that a 1 mm increase in precipitation decreased *T*_mean_ in July by 0.038°C during the period 1991–2015 (Y = −0.038*x* + 26.894, *R* ^2^ = .257, *p* = .010), which alleviated the influence of higher temperatures over 28°C on breeding. As mentioned above, precipitation also decreases soil temperatures and reduces the summer diapause of *H. armigera* (Hackett & Gatehouse, 1982), which could increase the viability of eggs; therefore, more moths could be produced. Additionally, during the period 1991–2015, the increase in precipitation increased the RH in July (Y = 0.215*x* + 40.089, *R* ^2^ = .334, *p* = .002), and the increase in RH would significantly decrease *T*_mean_ in July (Y = −0.143*x* + 32.510, *R* ^2^ = .502, *p* < .001) and increase the moth peak of G2 (Figure 5k). However, the increase in RH decreased Ln(relative abundance of G3) (Figure 5l). Thus, when we study the influence of precipitation on *H. armigera*, the suitable developmental temperatures and RH should be considered because precipitation has different impacts on the development of *H. armigera* during different periods (Li et al., 2016).

An abrupt change in temperature usually precedes an abrupt phenology change (Liu et al., 2007); however, all the abrupt changes observed in this study appeared after *T*_mean_ in June, except for the abrupt change in G2 moths. Additionally, the abrupt changes in G1 and G2 moths occurred far earlier than those in G3 and G1–G3 moths. This difference might suggest that their influencing mechanisms were different, and their primary affecting factors might support this hypothesis (Table 2). The phenological rate of change in insects is faster than that in plants under climate change (Gordo & Sanz, 2005; Parmesan, 2007). In this study, the abrupt changes in G1 and G2 moths were earlier than those in wheat and cotton, but the abrupt changes in G3 and G1–G3 moths were far later than those in wheat and cotton. The main hosts of G1 and G2 were wheat and cotton, respectively. The metabolism of insects is more sensitive to increases in temperature than that of plants (Bale et al., 2002; Berggren, Björkman, Bylund, & Ayres, 2009), and this sensitivity increases significantly with increasing trophic levels (Voigt et al., 2003). Climate abrupt change caused increases in the mean temperature, which would further affect the development, phenology, and numbers of *H. armigera*. The asynchrony between these changes might be increased due to climate warming. Thus, when analyzing population dynamics, it was necessary to consider abrupt climate change or changes in host phenology and (a)synchrony with moth phenology.

Crop varieties affect *H. armigera* populations. For example, moths of G2 decrease in number and may nearly vanish in high Bt cotton density regions, and moths of three generations (G1, G2, and G3) all show decreasing trends (Gao et al., 2010). Moths of the G0, G1, and G2 also decrease with an increase in the Bt cotton‐planted area in another study (Zhang, Ma, Xu, et al., 2013). However, in this study, the moths of three generations all increased, especially G3 moths (Figure 2b), because Bt cotton was not planted in this region. Bt cotton kills most of the larvae of G2 and cuts off the seasonal transfer chain of *H. armigera* and, thus, decreases the number of source larvae, alleviating harm to cotton and other crops (Lu et al., 2018). The function of Bt cotton in restraining the growth of *H. armigera* has not been applied in our study area, so it will be necessary to plant Bt cotton preventing the increases in *H. armigera*. The larvae of G1 mainly occur in wheat, and G1 moths oviposit in the buds or flowers of cotton in mid‐ and late May; some moths of G2 and G3 live on cotton, and others transfer to corn, which makes cotton fields the major source of subsequent *H. armigera* generations. It has been hypothesized that the greater the number of G2 moths, the more severe the harm to other crops at subsequent times (Guo, 1998). Our results provided support for this hypothesis: With an increase in G2 moths (Figure 2a), G3 moths dramatically increased (Figure 2b).

Host crops influence the abundance of *H. armigera* in many ways, such as affecting the suitability of the food supply and the availability of oviposition and pupation sites and refuges from natural enemies (Fitt, 1989; Kennedy & Storer, 2000; Sequeira, 2001). The growth stages of *H. armigera* vary according to their host plants, such as wheat, cotton, and maize (Liu et al., 2004), because main host of the first generation is wheat, while the second and third generations mainly use cotton and maize as hosts, respectively (Wu & Guo, 2005). This situation causes *H. armigera* abundance to change in different landscapes (Lu & Baker, 2013). For female moths, the choice of the type of host plant for oviposition shows no significant correlation with resultant offspring fitness (Jallow & Zalucki, 2003; Liu, Scheirs, & Heckel, 2012) because female moths can exploit different host plants in space and time through flight. A more complex agroecosystem can potentially maintain a more substantial population than that in a simple agroecosystem (Lu & Baker, 2013). In our study, 17 out of 25 years presented simple agriculture landscapes. Therefore, the *H. armigera* population dramatically fluctuated (Figure 2e). Different crop varieties, including wheat, cotton, and corn, were planted in this region during the past 25 years, which has resulted in different phenologies and planting dates and has served to enhance the abundance of *H. armigera*.

Crop planting percentages also impacts the *H. armigera* population (Lu et al., 2012). Our study showed that the number of moths exhibited a positive but insignificant correlation with the percentage of (non‐Bt) cotton (data not shown). Li et al. (2015) illustrated by using stable carbon isotope techniques (*δ* ^13^C) that approximately 50% of the moths that occurred in late May and between August and September came from C_4_ plants (such as corn), while approximately 100% of those that occurred in June and July came from C_3_ plants (such as wheat and cotton). In our study area, winter wheat was harvested by the end of the June, and cotton and corn were harvested in late September, so the G2 and G3 moths mainly came from cotton and corn, respectively. With an increase in the cotton‐ and corn‐planted areas (Figure 6h), G2 and G3 moths would increase (Figure 6a,b). However, the cotton‐planted area significantly increased by 1736.718 ha per year (*p* < .001), and the wheat‐ and corn‐planted areas also significantly increased, but only by 201.899 and 273.490 ha per year (*p* < .001), respectively. Thus, the percentages of the corn‐ and wheat‐planted areas showed significant decreasing trends (*p* < .001), while the percentage of the cotton‐planted area showed an increasing trend with an insignificant correlation (Figure 6g). Therefore, crop area and percentages should be considered when we study the relationships between crops and populations of *H. armigera*; otherwise, a unilateral conclusion might be drawn.

According to Lu et al. (2012), agricultural landscapes in which the cotton‐planted area is below or equal (≤) 50% of the total crop area are considered as “complex,” whereas where the area is over (>) 50%, they are considered as “simple.” In our study, 17 out of 25 years were defined as simple agricultural landscapes. The number of moths in a complex landscape is greater than that in a simple landscape because insects can feed on many kinds of plants to avoid a lack of food (Allen & Luttrell, 2009; Maelzer & Zalucki, 1999; Slosser, Witz, Puterka, Price, & Hartstack, 1987). Thus, we assume that the number of moths might increase with an increase in the complexity of landscapes.

## 5 CONCLUSIONS

Our results suggested that climate warming has advanced the phenology of G1, G2, and G3 moths and wheat and cotton flowering dates with different change rates, increasing the population numbers of *H. armigera*, especially G3 moths. For non‐Bt cotton landscapes, a complex landscape would increase adult moth numbers of *H. armigera*, and an increasing cotton‐planted area would steadily increase the moth numbers of *H. armigera*. Climate change has caused asynchronous changes between crops, *H. armiger*a, and climate factors. The asynchrony responses in crop flowering dates and phenology of *H. armigera* to climate warming would expand in the future. To reduce the increase in *H. armigrea* populations, Bt cotton should be planted, and efficient pest management is required.

## CONFLICT OF INTEREST

None declared.

## AUTHORS CONTRIBUTION

Jian Huang designed the experiment and conducted it, analyzed the data, and wrote the manuscript. Hongfei Hao conducted the experiment and analyzed the data.

## Data Availability

The data can be found in Figshare. https://figshare.com/account/home. https://doi.org/10.6084/m9.figshare.11328428.

## References

[ece35986-bib-0001] Allen, K. C. , & Luttrell, R. G. (2009). Spatial and temporal distribution of *Heliothines* and tarnished plant bugs across the landscape of an Arkansas farm. Crop Protection, 28(9), 722–727. 10.1016/j.cropro.2009.04.007

[ece35986-bib-0002] Bale, J. S. , Masters, G. J. , Hodkinson, I. D. , Awmack, C. , Bezemer, T. M. , Brown, V. K. , … Whittaker, J. B. (2002). Herbivory in global climate change research: Direct effects of rising temperature on insect herbivores. Global Change Biology, 8(16), 1–16. 10.1046/j.1365-2486.2002.00451.x

[ece35986-bib-0003] Berggren, Å. , Björkman, C. , Bylund, H. , & Ayres, M. P. (2009). The distribution and abundance of animal populations in a climate of uncertainty. Oikos, 118, 1121–1126. 10.1111/j.1600-0706.2009.17558.x

[ece35986-bib-0004] Berryman, A. , & Turchin, P. (2001). Identifying the density‐dependent structure underlying ecological time series. Oikos, 92, 265–270. 10.1034/j.1600-0706.2001.920208.x

[ece35986-bib-0005] Brook, B. W. , & Bradshaw, C. J. (2006). Strength of evidence for density dependence in abundance time series of 1198 species. Ecology, 87, 1445–1451. 10.1890/0012-9658(2006)87[1445:SOEFDD]2.0.CO;2 16869419

[ece35986-bib-0006] Chapman, J. W. , Drake, V. A. , & Reynolds, D. R. (2011). Recent insights from radar studies of insect flight. Annual Review of Entomology, 56, 337–356. 10.1146/annurev-ento-120709-144820 21133761

[ece35986-bib-0007] Chen, F. J. , Zhai, B. P. , & Zhang, X. X. (2003). Effects of soil moisture during pupal stage on population development of cotton bollworm, *Helicoverpa armigera* (Hübner). Acta Ecologica Sinica, 23(1), 112–121.

[ece35986-bib-0008] Chen, Y. S. , Chao, C. , & Liu, X. P. (2016). Photoperiod and temperature influence significantly diapause intensity of the cotton bollworm, *Helicoverpa armigera* (Lepidoptera: Noctuidae). Acta Entomologica Sinica, 56(2), 145–152.

[ece35986-bib-0009] Chown, S. L. , & Holter, P. (2000). Discontinuous gas exchange cycles in *Alhodius fossor* (Scarabaeidae): A test of hypotheses concerning origins and mechanisms. Journal of Experimental Biology, 203, 397–403. 10607549 10.1242/jeb.203.2.397

[ece35986-bib-0010] Coley, P. D. (1998). Possible effects of climate change on plant/herbivore interactions in moist tropical forests. Climatic Change, 39, 455–472.

[ece35986-bib-0011] Cornelissen, T. (2011). Climate change and its effects on terrestrial insects and herbivory patterns. Neotropical Entomology, 40(2), 155–163. 21584394 10.1590/s1519-566x2011000200001

[ece35986-bib-0012] Downes, S. , Kriticos, D. , Parry, P. , Paull, C. , Schellhorn, N. , & Zalucki, M. P. (2016). A perspective on management of *Helicoverpa armigera*: Transgenic Bt cotton, IPM, and landscapes. Pest Management Science, 73, 485–492. 10.1002/ps.4461 27753247

[ece35986-bib-0013] Field, A. (2000). Discovering statistics using SPSS for Windows. London, UK: Sage.

[ece35986-bib-0014] Fitt, G. P. (1989). The ecology of *Heliothis* species in relation to agroecosystems. Annual Review of Entomology, 34, 17–52. 10.1146/annurev.en.34.010189.000313

[ece35986-bib-0015] Franco, A. M. A. , Hill, J. K. , Kitschke, C. , Collingham, Y. C. , Roy, D. B. , Fox, R. , … Thomas, C. D. (2006). Impacts of climate warming and habitat loss on extinctions at species' low‐latitude range boundaries. Global Change Biology, 12(8), 1545–1553. 10.1111/j.1365-2486.2006.01180.x

[ece35986-bib-0016] Fu, C. B. , & Wang, Q. (1992). The definition and detection of the abrupt climatic change. Scientia Atmospherica Sinica, 16(4), 482–493.

[ece35986-bib-0017] Furlong, M. J. , & Zalucki, M. P. (2017). Climate change and biological control: The consequences of increasing temperatures on host‐parasitoid interactions. Current Opinion in Insect Science, 20, 39–44. 10.1016/j.cois.2017.03.006 28602234

[ece35986-bib-0018] Gao, K. H. , Zhang, J. , Liu, A. R. , & Wang, Q. X. (2007). Analysis on climatic conditions to occurrence of the second generation *Helicoverpa armigera* in Gaoqing county. Shandong Meteorology, 27(111), 53–54.

[ece35986-bib-0019] Gao, Y. L. , Feng, H. Q. , & Wu, K. M. (2010). Regulation of the seasonal population patterns of *Helicoverpa armigera* moths by *Bt* cotton planting. Transgenic Research, 19(4), 557–562. 10.1007/s11248-009-9337-1 19847665

[ece35986-bib-0020] Gao, Y. B. , & Zhai, B. P. (2010). Active temperature selection of flying *Helicoverpa armigera* (Lepidoptera: Noctuidae) moths. Acta Entomologica Sinica, 53(5), 540–548.

[ece35986-bib-0021] Ge, F. , Liu, X. H. , Ding, Y. Q. , Wang, X. Z. , & Zhao, Y. F. (2003). Life table of *Helicoverpa armigera* in Northern China and characters of population development in Southern and Northern China. Chinese Journal of Applied Ecology, 14(2), 241–245. 12827879

[ece35986-bib-0022] Gerstengarbe, F. W. , & Werner, P. C. (1999). Estimation of the beginning and end of recurrent events within a climate regime. Climate Research, 11, 97–107. 10.3354/cr011097

[ece35986-bib-0023] Gordo, O. , & Sanz, J. J. (2005). Phenology and climate change: A long‐term study in a Mediterranean locality. Oecologia, 146, 484–495. 10.1007/s00442-005-0240-z 16170564

[ece35986-bib-0024] Gu, S. , Han, P. , Ye, Z. , Perkins, L. E. , Li, J. , Wang, H. , … Lu, Z. (2018). Climate change favours a destructive agricultural pest in temperate regions; late spring cold matters. Journal of Pest Science, 91(4), 1191–1198. 10.1007/s10340-018-1011-z

[ece35986-bib-0025] Guo, J. Y. , Wan, F. H. , Hu, Y. H. , & Yan, Y. (2007). Effects of crop arrangement patterns on arthropod community structure in transgenic bollworm resistant cotton fields. Chinese Journal of Applied Ecology, 19(9), 2061–2068. 18062314

[ece35986-bib-0026] Guo, Y. Y. (1998). Studies on cotton bollworm. Beijing, China: China Agriculture Press.

[ece35986-bib-0027] Hackett, D. S. , & Gatehouse, A. G. (1982). Diapause in *Heliothis armigera* (Hübner) and *H. fletcheri* (Hardwick) (Lepidoptera: Noctuidae) in the Sudan Gezira. Bulletin of Entomological Research, 72, 409–422. 10.1017/s0007485300013584

[ece35986-bib-0028] Hance, T. , van Baaren, J. , Vernon, P. , & Boivin, G. (2007). Impact of extreme temperatures on parasitoids in a climate change perspective. Annual Review of Entomology, 52, 107–126. 10.1146/annurev.ento.52.110405.091333 16846383

[ece35986-bib-0029] Hetz, S. K. , & Bradley, T. J. (2005). Insects breathe discontinuously to avoid oxygen toxicity. Nature, 433, 516–519. 10.1038/nature03106 15690040

[ece35986-bib-0077] Höskuldsson, A. (1988). PLS regression methods. Journal of Chemometrics, 2(3), 211–228.

[ece35986-bib-0030] Huang, J. (2016). Different sowing dates affected cotton yield and yield components. International Journal of Plant Production, 10(1), 63–84.

[ece35986-bib-0031] Huang, J. , & Hao, H. F. (2018). Detecting mismatches in the phenology of cotton bollworm larvae and cotton flowering in response to climate change. International Journal of Biometeorology, 62, 1507–1520. 10.1007/s00484-018-1552-0 29752540

[ece35986-bib-0032] Huang, J. , & Li, J. (2017). Spring phenology of cotton bollworm affects wheat yield. Ecology and Evolution, 7, 1078–1090. 10.1002/ece3.2719 28303179 PMC5306014

[ece35986-bib-0033] Ives, A. R. , Paull, C. , Hulthen, A. , Downes, S. , Andow, D. A. , Haygood, R. , … Schellhorn, N. A. (2017). Spatio‐Temporal variation in landscape composition may speed resistance evolution of pests to Bt crops. PLoS ONE, 12(1), e0169167 10.1371/journal.pone.0169167 28046073 PMC5207666

[ece35986-bib-0034] Jallow, M. F. A. , & Zalucki, M. P. (2003). Relationship between oviposition preference and offspring performance in Australian *Helicoverpa armigera* (Hübner) (Lepidoptera:Noctuidae). Australian Journal of Entomology, 42, 343–348.

[ece35986-bib-0035] Kalyebi, A. , Sithanantham, S. , Overholt, W. , Hassan, S. A. , & Mueke, J. M. (2005). Parasitism, longevity and progeny production of six indigenous kenyan trichogrammatid egg parasitoids (hymenoptera: trichogrammatidae) at different temperature and relative humidity regimes. Biocontrol Science & Technology, 15(3), 255–270.

[ece35986-bib-0036] Kendall, M. G. (1948). Rank correlation methods. New York, NY: Hafner.

[ece35986-bib-0037] Kennedy, G. G. , & Storer, N. P. (2000). Life systems of polyphagous arthropod pests in temporally unstable cropping systems. Annual Review of Entomology, 45, 467–493. 10.1146/annurev.ento.45.1.467 10761586

[ece35986-bib-0038] Kreyling, J. (2010). Winter climate change: A critical factor for temperate vegetation performance. Ecology, 91, 1939–1948. 10.1890/09-1160.1 20715613

[ece35986-bib-0039] Kriticos, D. J. , Ota, N. , Hutchison, W. D. , Beddow, J. , Walsh, T. , Tay, W. T. , … Zalucki, M. P. (2015). The potential distribution of invading *Helicoverpa armigera* in North America: Is it just a matter of time? PLoS ONE, 10(3), e0119618 10.1371/journal.pone.0119618 25786260 PMC4364701

[ece35986-bib-0040] Kurz, W. A. , Dymond, C. C. , Stinson, G. , Rampley, G. J. , Neilson, E. T. , Carroll, A. L. , … Safranyik, L. (2008). Mountain pine beetle and forest carbon feedback to climate change. Nature, 452, 987–990. 10.1038/nature06777 18432244

[ece35986-bib-0041] Land, W. H. , Ford, W. , Park, J.‐W. , Mathur, R. , Hotchkiss, N. , Heine, J. , … Yeatman, T. (2011). Partial Least Squares (PLS) applied to medical bioinformatics. Procedia Computer Science, 6, 273–278. 10.1016/j.procs.2011.08.051

[ece35986-bib-0042] Li, H. , Yao, J. , Zhou, Q. , Wang, D. (2005). Effects of grain production systems on occurrence of second generation cotton bollworms in cotton fields in South Xinjiang. Xinjiang Agricultural Sciences. 42(2), 114–116.

[ece35986-bib-0043] Li, N. , Zhang, J. , Liu, Y. J. , Zhang, B. , Xiong, J. X. , Wang, P. L. , & Lu, Z. Z. (2015). Analysis of Larval host types of Cotton Bollworm (*Helicoverpa armigera*) Populations for Evaluation of Bt Refuges in Northern Xinjiang. Acta Ecologica Sinica, 35(19), 6280–6287.

[ece35986-bib-0044] Li, Z. , Zheng, Y. S. , & Tang, B. S. (2016). Study on the relationship between precipitation and number of cotton bollworm. Hubei Agricultural Sciences, 55(13), 3340–3348.

[ece35986-bib-0045] Liu, Y. F. , & Gu, D. X. (1997). An analysis of occurrence trends of crop pests with warming climate in China. Natural Enemies of Insects, 19(2), 93–96.

[ece35986-bib-0046] Liu, Z. D. , Gong, P. Y. , Wu, K. J. , & Li, D. M. (2004). Effects of high temperature on incidence of pupation, summer diapause and pupal weight of the cotton bollworm*, Helicoverpa armige*ra (Hübner). Acta Entomologica Sinica, 47(1), 14–19.

[ece35986-bib-0047] Liu, Z. , Gong, P. , Wu, K. , Wei, W. , Sun, J. , & Li, D. (2007). Effects of larval host plants on over‐wintering preparedness and survival of the cotton bollworm, *Helicoverpa armigera* (Hübner) (Lepidoptera:Noctuidae). Journal of Insect Physiology, 53, 1016–1026. 10.1016/j.jinsphys.2007.05.005 17597144

[ece35986-bib-0048] Liu, Z. D. , Scheirs, J. , & Heckel, D. G. (2012). Trade‐offs of host use between generalist and specialist *Helicoverpa* sibling species: Adult oviposition and larval performance. Oecologia, 168, 459–469. 10.1007/s00442-011-2103-0 21863244

[ece35986-bib-0049] Lu, Y. H. , Jiang, Y. Y. , Liu, J. , Zeng, J. , Yang, X. M. , & Wu, K. M. (2018). Adjustment of cropping structure increases the risk of cotton bollworm outbreaks in China. Chinese Journal of Aplied Entomology, 55(1), 19–24.

[ece35986-bib-0050] Lu, Z. Z. , & Baker, G. (2013). Spatial and temporal dynamics of *Helicoverpa armigera* (Lepidoptera, Noctuidae) in contrasting agricultural landscapes in northwestern China. International Journal of Pest Management, 59(1), 25–34.

[ece35986-bib-0051] Lu, Z. Z. , Pan, W. L. , Zhang, X. , Li, X. C. , & Zhang, J. (2012). The effect of cropping landscapes on the population dynamics of the cotton bollworm *Helicoverpa armigera* (Lepidoptera, Noctuidae) in the northern Xinjiang. Acta Ecologica Sinica, 32(24), 7925–7931.

[ece35986-bib-0052] Lu, Z. Z. , Zalucki, M. P. , Perkins, L. E. , Wang, D. Y. , & Wu, L. L. (2013). Towards a resistance management strategy for *Helicoverpa armigera* in Bt‐cotton in northwestern China: An assessment of potential refuge crops. Journal of Pest Science, 86, 695–703. 10.1007/s10340-013-0517-7

[ece35986-bib-0053] Lynch, M. , & Gabriel, W. (1987). Environmental tolerance. The American Naturalist, 129, 283–303. 10.1086/284635 16224689

[ece35986-bib-0054] Maelzer, D. A. , & Zalucki, M. P. (1999). Analysis of long‐term light‐trap data for *Helicoverpa* spp. (Lepidoptera: Noctuidae) in Australia: The effect of climate and crop host plants. Bulletin of Entomological Research, 89(5), 455–463.

[ece35986-bib-0055] Maelzer, D. A. , & Zalucki, M. P. (2000). Long range forecasts of the numbers of *Helicoverpa punctigera* and *H. armigera* (Lepidoptera:Noctuidae) in Australia using the Southern Oscillation Index and the sea surface temperature. Bulletin of Entomological Research, 90, 133–146. 10948373 10.1017/s0007485300000249

[ece35986-bib-0056] Maelzer, D. A. , Zalucki, M. P. , & Laughlin, R. (1996). Analysis and interpretation of long term light trap data for *Helicoverpa punctigera* (Lepidoptera: Noctuidae) in Australia: Population changes and forecasting pest pressure. Bulletin of Entomological Research, 86, 547–557.

[ece35986-bib-0057] Mann, H. B. (1945). Non‐parametric Test against Trend. Econometrika, 13, 245–259.

[ece35986-bib-0058] Meng, X. L. , Zhang, G. X. , & Ren, S. Z. (1962). Further study on biological characteristics of cotton bollworm. Acta Entomologica Sinica, 11(1), 71–82.

[ece35986-bib-0059] Miao, W. , Guo, Z. Y. , Lu, Z. Z. , Yu, J. N. , & Wang, D. Y. (2006). Estimation of generations of *Helicoverpa armigera* (Hübner) and *Aphis gossypii* (Glover) in Xinjiang based on single sine model. Xinjiang Agricultural Sciences, 43, 186–188.

[ece35986-bib-0060] Morton, R. , Tuart, L. D. , & Wardhaugh, K. G. (1981). The analysis and standardization of light‐trap catches of *Heliothis armigera* (Hübner) and *H. punctigera* Wallengren (Lepidoptera: Noctuidae). Bulletin of Entomological Research, 71, 207–225.

[ece35986-bib-0061] Naes, T. , & Martens, H. (1985). Comparison of prediction methods for multicollinearity data. Communications in Statistics—simulation and Computation, 14, 545–576.

[ece35986-bib-0062] Noor‐ul‐Ane, M. , Kim, D. S. , & Zalucki, M. P. (2018). Fecundity and egg laying in *Helicoverpa armigera* (Lepidotpera: Noctuidae): Model development and field validation. Journal of Economic Entomology, 111, 2208–2216. 10.1093/jee/toy183 29982457

[ece35986-bib-0063] Ouyang, F. , Hui, C. , Ge, S. , Men, X. Y. , Zhao, Z. H. , Shi, P. J. , … Li, B. L. (2014). Weakening density dependence from climate change and agricultural intensification triggers pest outbreaks: A 37‐year observation of cotton bollworms. Ecology and Evolution, 4(17), 3362–3374. 10.1002/ece3.1190 25535553 PMC4228611

[ece35986-bib-0064] Parmesan, C. (1996). Climate and species' range. Nature, 382, 765–766. 10.1038/382765a0

[ece35986-bib-0065] Parmesan, C. (2007). Influences of species, latitudes and methodologies on estimates of phenological response to global warming. Global Change Biology, 13(9), 1860–1872. 10.1111/j.1365-2486.2007.01404.x

[ece35986-bib-0066] Parmesan, C. , Ryrholm, N. , Stefanescu, C. , Hill, J. K. , Thomas, C. D. , Descimon, H. , … Warren, M. (1999). Poleward shifts in geographical ranges of butterfly species associated with regional warming. Nature, 399(6736), 579–583.

[ece35986-bib-0067] Parmesan, C. , & Yohe, G. (2003). A globally coherent fingerprint of climate change impacts across natural systems. Nature, 421(6918), 37–42. 12511946 10.1038/nature01286

[ece35986-bib-0068] Qin, J. D. (1964). Study on water in food and environment for *Mythimna seperata* larvae. Acta Entomologica Sinica, 13(5), 659–669.

[ece35986-bib-0069] Riis, L. , & Esbjerg, P. (1998). Movement, distribution, and survival of *cyrtomenus bergi* (hemiptera: cydnidae) within the soil profile in experimentally simulated horizontal and vertical soil water gradients. Environmental Entomology, 27(5), 1175–1181.

[ece35986-bib-0070] Russell, T. L. , Lwetoijera, D. W. , Knols, G. J. , Takken, W. , Killen, G. F. , & Ferguson, H. M. (2011). Linking individual phenotype to density‐dependent population growth: The influence of body size on the population dynamics of malaria vectors. Proceedings of the Royal Society B‐Biological Sciences, 278, 3142–3151. 10.1098/rspb.2011.0153 PMC3158942 21389034

[ece35986-bib-0071] Satake, A. , Ohgushi, T. , Urano, S. , & Uehimura, K. (2006). Modeling population dynamics of a tea pest with temperature‐dependent development: Predicting emergence timing and potential damage. Ecological Research, 21(1), 107–116. 10.1007/s11284-005-0099-9

[ece35986-bib-0072] Sequeira, R. (2001). Inter‐seasonal population dynamics and cultural management of *Helicoverpa spp*. in a Central Queensland cropping system. Australian Journal of Experimental Agriculture, 41, 249–259.

[ece35986-bib-0073] Sibly, R. M. , Barker, D. , Denham, M. C. , Hone, J. , & Pagel, M. (2005). On the regulation of populations of mammals, bird, fish, and insects. Science, 309, 607–610. 16040705 10.1126/science.1110760

[ece35986-bib-0074] Slosser, J. E. , Witz, J. A. , Puterka, G. J. , Price, J. R. , & Hartstack, A. W. (1987). Seasonal changes in bollworm (Lepidoptera: Noctuidae) moth catches in pheromone traps in a large area. Environmental Entomology, 16(6), 1296–1301. 10.1093/ee/16.6.1296

[ece35986-bib-0075] Standardization Administration of the People's Republic of China . (2009). Rules for investigation and forecast of the cotton bollworm [*Helicoverpa armigera* (Hübner)](GB/T 15800–2009).

[ece35986-bib-0076] Tauber, M. J. , Tauber, C. A. , Nyrop, J. P. , & Villani, M. G. (1998). Moisture, a vital but neglected factor in the seasonal ecology of insects: Hypotheses & tests of mechanisms. Environmental Entomology, 27, 523–530.

[ece35986-bib-0078] Thomas, C. D. , Cameron, A. , Green, R. E. , Bakkenes, M. , Beaumont, L. J. , Collingham, Y. C. , … Williams, S. E. (2004). Extinction risk from climate change. Nature, 427, 145–148. 10.1038/nature02121 14712274

[ece35986-bib-0079] Turchin, P. (1999). Population regulation: A synthetic view. Oikos, 84, 153–159. 10.2307/3546876

[ece35986-bib-0080] Voigt, W. , Perner, J. , Davis, A. J. , Eggers, T. , Schumacher, J. , Bährmann, R. , … Sander, F. W. (2003). Trophic levels are differentially sensitive to climate. Ecology, 84, 2444–2453. 10.1890/02-0266

[ece35986-bib-0081] Wardhaugh, K. G. , Room, P. M. , & Greenup, L. R. (1980). The incidence of *Heliothis armigera* (Hübner) and *H. punctigera* Wallengren (Lepidoptera: Noctuidae) on cotton and other host‐plants in the Namoi Valley of New South Wales. Bulletin of Entomological Research, 70, 113–131.

[ece35986-bib-0082] Wei, F. Y. (1999). Modern technology of statistics, diagnosis and forecast for climate (pp. 62–76). Beijing: China Meteorological Press.

[ece35986-bib-0083] Westgarth‐Smith, A. R. , Leroy, S. A. G. , Collins, P. E. F. , & Harrington, R. (2007). Temporal variations in English populations of a forest insect pest, the green spruce aphid (Elatobium abietinum), associated with the North Atlantic Oscillation and global warming. Quaternary International, 173‐174, 153–160. 10.1016/j.quaint.2007.05.001

[ece35986-bib-0084] Wolf, W. W. , Westbrook, J. K. , Raulston, J. , Pair, S. D. , & Hobbs, S. E. (1990). Recent airborne radar observations of migrant pests in the United States. Philosophical Transactions of the Royal Society B: Biological Sciences, 328, 619–630.

[ece35986-bib-0085] Wu, K. M. , & Guo, Y. Y. (1996). Flight activity in *Helicoverpa armigera* . Acta Ecologica Sinica, 16(6), 612–617.

[ece35986-bib-0086] Wu, K. M. , & Guo, Y. Y. (2000). On the cold hardiness of cotton bollworm populations from Xinjiang uygur autonomous region. Acta Phytopathologica Sinica, 27(1), 23–26.

[ece35986-bib-0087] Wu, K. M. , & Guo, Y. Y. (2005). The evolution of cotton pest management practices in China. Annual Review of Entomology, 50, 31–52. 10.1146/annurev.ento.50.071803.130349 15355239

[ece35986-bib-0088] Wu, K. M. , Lu, Y. H. , Feng, H. Q. , Jiang, Y. Y. , & Zhao, J. Z. (2008). Suppression of cotton bollworm in multiple crops in China in areas with Bt toxin‐containing cotton. Science, 321(5896), 1676–1678. 18801998 10.1126/science.1160550

[ece35986-bib-0089] Wu, Y. (1999). Analysis of affecting factors on cotton bollworm occurrence in Liaoyang region. Plant Protection, 25(4), 24–26.

[ece35986-bib-0090] Xu, G. , Guo, Y. , Wu, K. , & Jiang, J. (2000). On the mark‐release techniques of Helicoverpa armigera (Hübner). Acta Gossypii Sin., 12, 247–250.

[ece35986-bib-0091] Xu, W. N. , & She, W. M. (1980). China agricultural meteorological observation guidelines (p. 212). Beijing, China: China Meteorology Press.

[ece35986-bib-0092] Yan, M. H. , Deng, W. , & Chen, P. Q. (2003). Analysis of climate jumps in the Sanjiang Plain. Scientia Geographica Sinica, 23(6), 661–667.

[ece35986-bib-0093] Zhai, L. R. , Ding, Y. Q. , & Li, D. M. (1992). Studies on the foraging behavior of *Heliothis armigera* (Hübner) and damaged fruiting structures in cotton fields of north china. Acta Entomologica Sinica, 35(3), 257–266.

[ece35986-bib-0094] Zhang, D. , Li, H. , Ma, L. Y. , & Guo, D. K. (2000). Research of predicting models on occurrence of the fifth generation cotton bollworm. Shanghai Agricultural Science and Technology, 33(2), p48.

[ece35986-bib-0095] Zhang, J. , Ma, J. H. , Xu, Y. C. , Wang, X. , Wang, P. L. , Wumaier, G. , & Lu, Z. Z. (2013). Migration behavior of cotton bollworm in Xinjiang of Northwest China based on the ovarian development characteristics of adult females. Chinese Journal of Ecology, 32(6), 1428–1432.

[ece35986-bib-0096] Zhang, X. W. , & Zhang, J. B. (2006). Xinjiang meteorology manual. Beijing, China: Meteorology Publishing.

